# Effect of Salvia officinalis scent on postmenopausal women’s sexual function and satisfaction: a randomized controlled trial

**DOI:** 10.1186/s12905-023-02605-8

**Published:** 2023-08-23

**Authors:** Sousan Heydarpour, Foruzan Sharifipour, Fateme Heydarpour

**Affiliations:** 1 Department of Reproductive Health, Nursing and Midwifery School, Kermanshah University of Medical Sciences, Kermanshah, Iran; 2 Department of Midwifery, Nursing and Midwifery School, Kermanshah University of Medical Sciences, Kermanshah, Iran; 3 Social Development & Health Promotion Research Center, Health Institute, Kermanshah University of Medical Sciences, Kermanshah, Iran

**Keywords:** Aromatherapy, Salvia officinalis, Sexual satisfaction, Sexual function, Menopausal women

## Abstract

**Introduction:**

Sexual function is one of the important aspects of quality of life which is often impaired after menopause. Given the side effects of hormone therapy on postmenopausal women, alternative treatments such as aromatherapy have won popularity. The aim of this study was to investigate the effect of aromatherapy using Salvia officinalis on the sexual function and satisfaction of postmenopausal women.

**Methods:**

This was a double-blind randomized controlled trial conducted on postmenopausal women referring to health centers in Kermanshah, Iran, in 2018. The participants were randomly divided into two groups: Salvia officinalis (n = 32) and control (n = 32). Women in the intervention group received 2 drops of Salvia officinalis essential oil by inhalation twice a day for 5 consecutive days of a week continued for 6 weeks. The control group received almond oil in the same dosage and frequency. Sexual function and satisfaction were evaluated using the Lindberg sexual satisfaction questionnaire and the female sexual function index, respectively before the intervention and 6 weeks after it.

**Results:**

After 6 weeks of intervention, the total mean scores of sexual function (28.8 ± 2.13 vs.17.9 ± 1.59 P < 0.001) and sexual satisfaction (71.53 ± 5.86 vs. 50.44 ± 10.41) were significantly higher in the Salvia officinalis group compared with the control group, respectively.

**Conclusion:**

The findings showed that aromatherapy using Salvia officinalis has a significant effect on improving sexual function and satisfaction in postmenopausal women. Therefore, given the prevalence of sexual disorders in postmenopausal women, aromatherapy using Salvia officinalis is recommended to be used for improving these disorders.

**Clinical trial registration:**

Iranian Registry of Clinical Trials; https://en.irct.ir/user/trial/50212/view (IRCT20160427027633N6), registered (12/08/2020).

## Introduction

Sexual function is an important part of life and the satisfaction obtained from it, is one of the most basic aspects of women’s life [1]. Today, the role of sexual function in the quality of life has been considered as an important issue in health care, so that sexual dysfunction can have a detrimental effect on the quality of life of women [2]. Menopause is one of the most important factors that can affect sexual performance [3]. The physical and mental changes associated with menopause are of paramount importance and one of the important issues in the field of reproductive health [4]. The most significant symptom of menopause is the end of fertility and cessation of menstruation, which occurs due to the inactivity of ovarian follicles and the decreased level of estrogen [5]. Due to the increase in life expectancy in the world population, women live longer after menopause [6]. The population of postmenopausal women around the world is expected to reach 1.1 billion by 2025, and in Iran this number will reach nearly 5 million [7]. During menopause, lower levels of ovarian hormones, especially estrogen, can lead to multiple physical, psychological, and sexual complications [8]. The decreased levels of estrogen and androgen during menopause lead to decreased blood flow in the vulva and vagina, and this will in turn result in the manifestation of genitourinary symptoms such as vaginal atrophy and dryness, dyspareunia, and a decrease in mental-sexual energy. These symptoms that are often ignored are one of the factors influencing sexual function and satisfaction [9].

Sexual function is an indispensable part of human life and behavior and is so interwoven with a person’s personality that it seems impossible to talk about it as an independent phenomenon [10]. Sexual dysfunction is defined as a persistent or recurring decrease in sexual arousal, dyspareunia, and difficulty or inability to reach orgasm, which can affect the quality of life [11]. Sexual satisfaction is an emotional response resulting from a person’s evaluation of their sexual relationship and includes satisfaction of the individual’s sexual needs, fulfillment of the expectations of the individual and their sexual partner, and the individual’s positive evaluation of the entire sexual relationship [12]. The results of a 2018 study on women in the United States showed that sexual problems increase with age, and disorders occur in the age range of 18–44 (27.2%) and 45–64 years (44.6%) [13]. In Iran, the relative frequency of sexual dysfunction reported by postmenopausal women is 65.2%. The most common sexual disorders are related to domains of desire (86%, n: 141), arousal (82.3%, n: 135), lubrication (71.3%, n: 117) and sexual satisfaction (70.1%, n: 115) [14].

The various methods that are commonly used to prevent complications associated with menopause and improve the quality of sexual life during menopause are generally divided into two groups: complementary and alternative medicine (CAM) and hormone therapy [15]. Side-effects associated with hormone therapy include gastrointestinal complications, weight gain, increased risk of breast and endometrial cancer, heart disease, thromboembolism, and liver adenoma [16]. The commonly reported adverse effects of hormone therapy led to a shift towards CAM. Aromatherapy is one of CAM practices which essentially involves the use of oils and extracted aromatic plant substances through inhalation or topical administration with bathing, acupressure, and massage [17]. The therapeutic properties of this approach are effective through psychological and physiological pathways (such as amygdala and hippocampus) [18].

Considering that estrogen deficiency contributes to menopausal complications, the use of herbal estrogens can be effective in reducing some menopausal symptoms. Phytoestrogens are natural nonsteroidal phenolic plant compounds that have estrogenic properties [19]. Salvia officinalis as a member of the Lamiaceae family is one of these phytoestrogens that is used as a medicinal plant in Iranian medicine. It contains alkaloids, flavonoids, phenolic acids, tannins and toogens [20]. In addition to its estrogenic, antibiotic, anti-spasmodic, anti-anxiety, anti-fungal, and anti-toxic properties, Salvia officinalis has been reported to reduce blood sugar [21].

Previous studies have shown that aromatherapy has a significant effect on improving sexual function and quality of life of postmenopausal women [15, 22, 23]. According to a systematic review, combined aromatherapy (neroli, lavender, fennel, geranium, and rose oil) results in significant improvement of sexual function in postmenopausal women [24]. Based on previous studies, Salvia officinalis extract improves menopausal symptoms in postmenopausal women [25, 26].

Given the importance of sexual health in postmenopausal women and the relatively high prevalence of sexual dysfunction among them, it is necessary to use non-pharmacological interventions for its management since they have low complications. To the best of our knowledge, no study has yet evaluated the effect of Salvia officinalis scent on the sexual function and satisfaction of postmenopausal women. Therefore, this study was conducted to investigate the effect of aromatherapy using Salvia officinalis on the sexual function and satisfaction of postmenopausal women.

## Methods

### Study design and participants

**Fig. 1:**
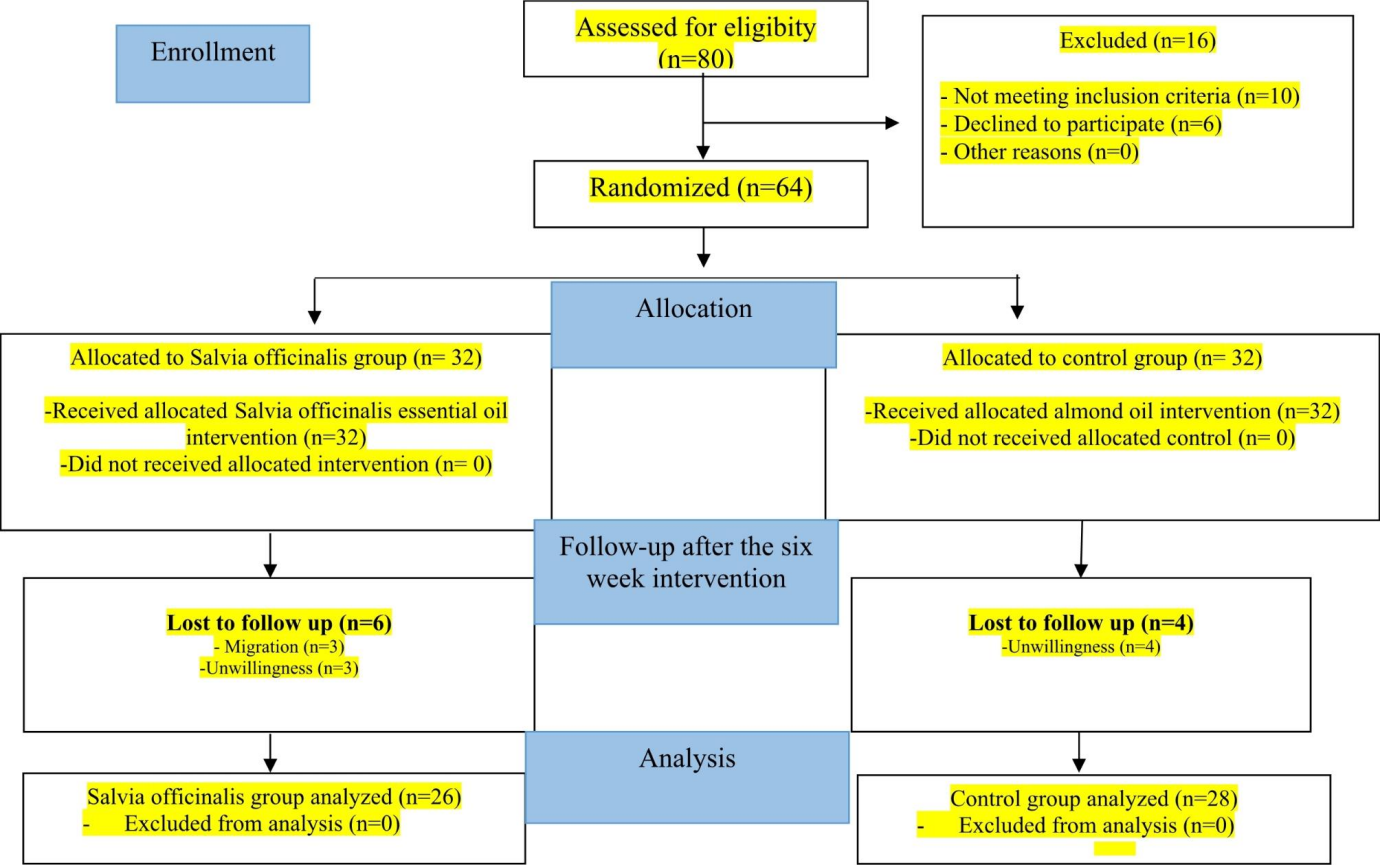
Flowchart of study participants

This double-blind randomized controlled clinical trial (RCT) using two parallel groups was conducted on eligible postmenopausal women referring to health centers in Kermanshah, Iran, from April to September 2019. Figure 1 shows the CONSORT flowchart of the study. Inclusion criteria were: age 45 to 60 years, menopause for at least 12 months, ability to read and write, FSFI score ≤ 28, being married and living with a husband, not having mental or physical illness, not smoking, no alcohol consumption, no history of allergic rhinitis or known respiratory problems such as asthma, no experience of stressful events (such as divorce, death of first-degree family members, etc.) during the 6 months prior to the study, and having a health record at the health care center. The exclusion criteria were unwillingness to continue participation and not completing the intervention for any reason.

### Sample size calculation

The sample size for each group was calculated based on the results of a previous similar study [22]. The following formula was used to calculate the sample size.

$$N=\frac{({{Z}_{1-\frac{\alpha }{2}}+{Z}_{1-\beta })}^{2} \times ({s}_{1}^{2}+{s}_{2}^{2})}{{({\mu }_{1}- {\mu }_{2})}^{2}}$$

Where α = 0.01, β = 0.1, µ1 = 1.4, µ2 = 4.4, S1 = 4.2, and S2 = 0.4, and n = 31. We added 5% for the dropout and the total number of women in each group was calculated to be 32.

### Sampling and random allocation method

After approval was obtained from the Ethics Committee of Kermanshah University of Medical Sciences, four health centers in Kermanshah were designated for data collection. Eligible postmenopausal women were randomly assigned to Salvia officinalis (n = 32) and control (n = 32) groups and completed the baseline test questionnaires. Women were taught how to use aromatherapy. Each week, one of the researchers made phone calls to the participants to ensure proper use of the oil and to check for any adverse effects. After the six-week intervention, post-tests were conducted including the same questionnaires as in the baseline test to determine the effects of the intervention. In total, data was collected from two time points.

Permuted block randomization method was used in this research. The allocation sequence was determined by the computer using a table of random numbers with a block size of 6 and an allocation ratio of 1:1. Salvia officinalis and almond oils were stored in identical containers and coded by a pharmacist. To blind the allocation, a unique code was assigned to each woman and all codes were kept in an opaque envelope until the intervention. Neither the statistician (who performed the data analysis) nor the researcher (who participated in the sampling) was aware of group allocation.

### Aromatherapy intervention

The women in the intervention and control groups were asked to put 2 drops of 10% Salvia officinalis or almond oil essence, respectively on the skin of their forearm (left or right) twice a day (10 am and 10 pm) and inhale the scent for 5 consecutive days per week, continued for 6 weeks. Following Malakouti et al., we asked the participants to sit in a comfortable position and place their forearm at a distance of 30 cm from their nose and inhale the scent for five minutes with normal breathing [22]. Essential oil of Salvia officinalis 10% was purchased from a pharmaceutical research center in Tehran (Iran) and approved by the Faculty of Pharmacy, Kermanshah University of Medical Sciences. Odorless almond oil diluted with propylene glycol was purchased from the herbal market in Kermanshah (Iran) and approved by the Faculty of Pharmacy, Kermanshah University of Medical Sciences. Based on previous studies, almond oil was used in the control group [27, 28]. The concentration of essential oil was 10%, i.e. 10 mg of Salvia officinalis essential oil in 100 ml of odorless almond oil diluted with propylene glycol. Propylene glycol maintained the stability of the essential oil. The amount of essential oil needed for a 6-week intervention was stored in a dropper by a pharmacist and given to the participants at the beginning of the study.

### Data collection tools

#### Demographic-social information form

This form was used to record information such as age, menopause age, educational attainment, occupation, monthly income, body mass index, and number of children.

#### Female sexual function index (FSFI)

This questionnaire included 19 questions scored based on a scale from 0 to 5 or 1 to 5, where 0 indicated no sexual activity in the last month (Table 1) [29]. Scores were standardized for each of the six domains by adding individual domain search scores and multiplying it by the domain factor. For example, 0.6 for desire, 0.3 for arousal and lubrication, and 0.4 for orgasm, satisfaction, and pain. The total FSFI score is the sum of the scores of six separate domains [30]. The range of the total score was between 2 and 36, a higher score indicates better sexual performance. Less than 28 was considered sexual dysfunction [31]. Rosen et al. confirmed the validity and reliability of the FSFI, with the average reliability of the whole scale being 0.88 and that of the domains ranging from 0.79 to 0.8620 [29]. In Iran, it has been confirmed by Mohammadi et al. (2007) on 53 women with a sensitivity of 83% and a specificity of 82% [31].

**Table 1:** Female Sexual Function Index scoring system. A domain score of zero indicates that no sexual activity was reported during the past month

Domain	Questions	Score range	Factor	Minimum score	Maximum score
Desire	1,2	1–5	0.6	1.2	6
Arousal	3,4,5,6	0–5	0.3	0	6
Lubrication	7,8,9,10	0–5	0.3	0	6
Orgasm	11,12,13	0–5	0.4	0	6
Satisfaction	14,15,16	0 (or1)-5	0.4	0	6
Pain Reduction	17,18,19	0–5	0.4	0	6
Full scale score range	-	-	-	2	36

*Lindaberg’s Sexual Satisfaction Questionnaire*: This questionnaire included 17 questions that are scored as follows: always: 5, often: 4, sometimes: 3, rarely: 2, and never: 1. The maximum and minimum scores are 85 and 17, respectively. Scores between 17 and 51 represent poor satisfaction, 67 − 52 suggest moderate satisfaction, and 85 − 65 show good satisfaction. This questionnaire was prepared by Lindaberg in 1997, and its validity and reliability were confirmed in Iran by Salehi Federdi. The reliability of the interview form and average sexual satisfaction were confirmed by the inter-rater reliability by obtaining correlation coefficients of r < 0.7 and r = 0.78, respectively, and a Cronbach’s alpha coefficient of 0.87 was obtained [32, 33].

### Statistical analysis

All data were analyzed using SPSS version 23. Statistical significance level was set at P < 0.05. Mean and standard deviation were used to describe quantitative variables while percentage and frequency were used to describe qualitative variables. The normal distribution of numerical variables was checked using the Kolmogorov-Smirnov test. Independent t-test or Mann-Whitney test was used to examine the difference between two groups. Paired t-test or its non-parametric equivalent (Wilcoxon test) was used to evaluate within-group differences. Socio-demographic characteristics of the studied women were compared using independent t-test, Mann-Whitney test, and Fisher’s exact test.

## Results

Initially, 64 women participated in this study. However, 6 women in the Salvia officinalis group and 4 in the control group were excluded from the study for reasons that are listed in Fig. 1.

Table 2 shows the socio-demographic characteristics of the participants in the Salvia officinalis and almond oil groups. The mean age of the participants was 58.59 ± 1.5 years, and their mean age of menopause was 52.01 ± 2.67 years. No significant difference was observed between the two groups in terms of demographic characteristics (P > 0.05).

**Table 2:** Socio-demographic characteristics of Salvia officinalis and control groups

Characteristics of participants		Salvia officinalis n = 26 Mean ± SD Or N (%)	control n = 28 Mean ± SD Or N (%)	P -valve
^Age(year)^		57.80 ± 4.98	59.39 ± 5.23	^T=1.138, p−value=0.261^
Menopausal age (year)		51.88 ± 2.43	52.15 ± 2.91	^T=2.010, p−value=0.061^
The duration of menopause (month)		77.61 ± 65.22	72.64 ± 61.34	^T=0.289, p−value=0.774^
Body mass index (cm/m²)		28.43 ± 4.35	27.45 ± 3.87	^T=0.882, p−value=0.382^
Education level	Less than diploma	15(57.7)	(60.70)17	^Fisher^ ^exact=0.745 p−value=0.299^
	diploma	8(30.8)	7(25)	
	university	3(11.5)	4(14.3)	
Number of children	0–2	3(11.5)	5(17.8)	^Fisher^ ^exact=0.852 p−value=0.517^
	3–4	17(65.4)	15(53.6)	
	> 5	6(23/1)	8(28.6)	
Monthly income	weak	4(15.4)	6(21.4)	^Fisher^ ^exact=0.706 p−value=0.431^
	moderate	17(65.4)	13(46.4)	
	good	5(19,2)	9(32.1)	
Occupational status	employee	3(11.5)	5(17.8)	^Fisher^ ^exact=0.588 p−value=0.274^
	homemaker	23(88.5)	23(82.2)	

Table 3 shows the mean total scores of FSFI in the Salvia officinalis and control groups at the baseline, and no statistically significant difference was observed between the two groups (15.96 ± 1.71 vs. 15.59 ± 1.44, respectively, P = 0.39). However, after intervention, the mean ± standard deviation of FSFI scores in the Salvia officinalis group was significantly higher than that in the control group (28.28 ± 2.13 vs. 17.90 ± 1.59, respectively, P < 0.001). Based on between-group analysis, women who received Salvia officinalis scent had significantly greater improvement in their total FSFI score and scores of all six domains compared to women who received almond oil (P < 0.001). According to the within-group analysis based on Wilcoxon results, there was no significant difference before and after the intervention in the control group in terms of the FSFI scores (P = 0.19), while there was a significant difference before and after the intervention in the Salvia officinalis group (P < 0.001). After 6 weeks of intervention, the total scores of FSFI and its domains in the Salvia officinalis group were significantly higher than those in the control group (P < 0.001).

**Table 3:** Mean of FSFI total and domains score in the Salvia officinalis and control groups at before and after intervention

Variables	Study group	Stage of intervention		Wilcoxon test P - value (within groups)
		Before Mean ± SD	After Mean ± SD	
**FSFI total score** Mann-Whitney U test P-value (between groups)	Salvia officinalis control	15.96 ± 1.71 15.59 ± 1.44 0.39	28.28 ± 2.13 17.90 ± 1.59 < 0.001	< 0.001 0.19
**Desire** Mann-Whitney U test P-value (between groups)	Salvia officinalis control	2.83 ± 0.76 2.80 ± 0.61 0.87	5.12 ± 0.64 2.74 ± 0.62 < 0.001	< 0.001 0.58
**Arousal** Mann-Whitney U test P-value (between groups)	Salvia officinalis control	2.50 ± 0.65 2.62 ± 0.49 0.63	1.20 ± 0.51 2.50 ± 0.0.55 < 0.001	< 0.001 0.12
**Lubrication** Mann-Whitney U test P-value (between groups)	Salvia officinalis control	2.55 ± 0.63 2.45 ± 0.47 0.81	4.68 ± 0.60 2.60 ± 0.56 < 0.001	< 0.001 0.70
**Orgasm** Mann-Whitney U test P-value (between groups)	Salvia officinalis control	2.61 ± 0.57 2.74 ± 0.99 0.27	4.49 ± 0.59 2.9 ± 0.63 < 0.001	< 0.001 0.23
**Pain Reduction** Mann-Whitney U test P-value (between groups)	Salvia officinalis control	2.63 ± 0.61 2.88 ± 0.65 0.46	4.64 ± 0.63 2.91 ± 0.66 < 0.001	< 0.001 0.13
**Satisfaction** Mann-Whitney U test P-value (between groups)	Salvia officinalis control	2.68 ± 0.55 2.55 ± 0.43 0.22	4.69 ± 0.56 4.67 ± 0.48 < 0.001	< 0.001 0.38

Table 4 shows the mean scores of sexual satisfaction in the Salvia officinalis group and the control group at the baseline, and no statistically significant difference was observed between the two groups (46.00 ± 10.06 vs. 48.35 ± 13.73, respectively, P = 0.89). However, after the intervention, the mean ± standard deviation of sexual satisfaction scores in the group receiving Salvia officinalis was significantly higher than that in the control group (71.53 ± 5.86 vs. 50.44 ± 10.41, respectively, P < 0.001). According to within-group analysis based on the results of the paired t test, there was no significant difference before and after the intervention in the control group in terms of their satisfaction scores (P = 0.18). However, there was a significant difference between the satisfaction scores of the Salvia officinalis group before and after the intervention (P < 0.001). After 6 weeks of intervention, the mean score of sexual satisfaction in the Salvia officinalis group was significantly higher than that of the control group (P < 0.001). Compared to the control group, the Salvia officinalis group did not show any significant adverse effects.

**Table 4:** Mean of sexual satisfaction score in the Salvia officinalis and control groups at before and after intervention

Variables	Study group	Stage of intervention		paired t-test P-value (within groups)
		**Before Mean ± SD**	**After Mean ± SD**	
**Sexual satisfaction score** Independent t-test P-value (between groups)	Salvia officinalis control	46.00 ± 10.06 48.35 ± 13.73 0.89	71.53 ± 5.86 50.44 ± 10.41 < 0.001	< 0.001 0.18

## Discussion

The aim of this study was to investigate the effect of aromatherapy using Salvia officinalis on the sexual function and satisfaction of postmenopausal women. The results of the present study showed that after the intervention, the mean score of FSFI and all its dimensions in the group receiving Salvia officinalis essential oil were significantly higher than those in the control group. These results show that aromatherapy with Salvia officinalis improves sexual function in postmenopausal women. Malakouti et al. (2016) conducted a clinical trial on 120 postmenopausal women to investigate the effect of inhalation aromatherapy on the sexual function of postmenopausal women. The intervention group received 2 to 3 drops of an aromatic solution (lavender, fennel, geranium, and rose) on the forearm three times a day for 6 weeks while the control group received a placebo containing propylene glycol as often as the intervention group. The findings showed that the overall score of sexual function in the two intervention and control groups was 18 ± 5.4 and 15.8 ± 5.7, respectively. However, after the intervention, these scores rose to 22.9 ± 0.4 in the intervention group and to 17.2 ± 4.2 in the placebo group (P˃0.001). Based on their results, there was a statistically significant difference between the aromatherapy and control groups in terms of the overall score of sexual function [22], which was consistent with the results of the present study. However, in the present study, only Salvia officinalis essential oil was used.

Aromatherapy through plant essential oils can be used in a number of ways. This can be done by inhaling the odor molecules (the sense of smell) and sending impulses to the olfactory part of the brain (which interacts with other control systems such as memory, emotions, hormones, sexual feelings, and heart rate), which will release hormones capable of stimulating, incubating, calming or inducing euphoria. Volatile oils are also absorbed by the skin and subsequently penetrate into the bloodstream [24]. Aromatherapy has been reported to increase libido and reduce anxiety and depression by reducing the release of stress hormones and increasing beta endorphins in the body [34]. The literature is scanty with regard to studies examining the role of aromatherapy in sexual function and satisfaction.

As one of the plants containing phytoestrogens (estrogen-like compounds), Salvia officinalis has a special place among plants used for the treatment of menopause symptoms. These plants can also be used to improve sexual function and dyspareunia and increase sexual satisfaction of menopausal women [35]. Phytoestrogens (plant compounds) are nonsteroidal polyphenolic compounds with 1 to 3 hydroxyl groups (OH) similar to the hydroxyl group in the phenolic estrogen circle [36]. Thus, low postmenopausal estrogen levels are partially compensated by phytoestrogens, bringing about improved menopausal symptoms [37].

The results of a clinical trial study by Dadfar and Bamdad (2019) showed that Salvia officinalis extract is effective in reducing the severity of some menopausal symptoms in postmenopausal women [26]. In their study, the participants received a 100 mg capsule of Salvia officinalis extract daily for 4 weeks. Dadfar and Bamdad suggested that Salvia officinalis extract may be an effective treatment for menopausal symptoms such as hot flashes, night sweats, panic attacks, fatigue, and concentration, which is consistent with the findings of the present study. Of course, their study was different from ours in that Saliva officinalis was used orally to evaluate its effect on the severity of general symptoms of menopause in postmenopausal women. The results of another study showed that Salvia officinalis is clinically effective in treating menopause-related symptoms such as hot flashes, night sweats, palpitations, muscle and joint pain, depression, anxiety, sleep disorders, and libido problems [25]. Despite the differences in design, duration, and method of intervention as well as the type of questionnaires used, the results of our study are consistent with these results.

The results of the present study showed that after the intervention, the mean score of sexual satisfaction in the group receiving Salvia officinalis essential oil were significantly higher than those in the control group. These results show that aromatherapy with Salvia officinalis improves sexual satisfaction in postmenopausal women. Sharifipour et al. (2023) conducted a clinical trial on 180 breastfeeding women to investigate the effect of inhalation aromatherapy on the sexual satisfaction of breastfeeding women. The participants were allocated to three groups of Citrus aurantium (n = 60), Lavender (n = 60), and control (n = 60) groups. Two intervention groups received 2 drops of essential oil, twice a day, for 40 days as inhalation while the control group received almond oil in the same. The findings showed that after the 40 days of intervention, the mean score of sexual satisfaction was significantly higher in the Citrus aurantium and Lavender groups compared with the control group (59.3 ± 11.7, 59.3 ± 11.6 vs. 52.02 ± 11.5, p < 0.001). Based on their results, the aroma of Citrus aurantium and Lavender essence could significantly improve the sexual satisfaction of breastfeeding women [38]., which was consistent with the results of the present study. However, in the present study, the participants were postmenopausal women who used Salvia officinalis essential oil.

The results of another trial aimed at determining the effect of combined aromatherapy on sexual function showed the effectiveness of this method. However, it had no effect on dyspareunia and sexual satisfaction [39], which can be attributed to other factors such as sexual knowledge and psychological factors, since the sexual function of postmenopausal women is affected by an amalgam of factors.

The results of Nikjou et al.‘s (2018) study showed that lavender inhalation for 20 min twice a day for 12 weeks improved libido among postmenopausal women with complaints of hot flashes [15], which is consistent with the results of the present study. However, the participants in the present study did not have any mental or physical diseases and inhaled 2 drops of Salvia officinalis essential oil twice a day for 5 consecutive days a week which continued for 6 weeks.

The first of the limitations of this study was that despite the training on how to use aromatherapy and the weekly follow-up by telephone, it is possible that the study subjects received different doses of the inhaler. The second of the limitations of this study was that data collection was based solely on participants’ own reports, and the researchers did not use other sources of data such as observation. The third limitation was that we did not assess the causes and duration of sexual dysfunction since these could affect women’s sexual function and satisfaction. Furthermore, we only studied the short-term effects of aromatherapy. On the other hand, this study is worthwhile in that it is the first to evaluate the effect of Salvia officinalis scent on the sexual function and satisfaction of postmenopausal women. Also, random allocation and concealment of allocation of subjects to prevent selection bias and using a blind method to reduce the risk of bias during data collection were other strengths of the study.

## Conclusion

Based on the results of the present study, aromatherapy with Salvia officinalis improves the sexual function and satisfaction of postmenopausal women and does not have any side effects. When providing counseling services to menopausal women to alleviate their sexual problems, health care providers should be aware of the benefits of Salvia officinalis in reducing sexual dysfunction and recommend using it. However, more clinical trials with longer follow-up periods on participants with physical and mental illnesses are needed to confirm these findings.

## Data Availability

The data that support the findings of this study are available from the corresponding author upon reasonable request.

## Abbreviations

CAM: complementary and alternative medicine
RCT: randomized controlled clinical trial
FSFI: Female sexual function index
